# Seroepidemiology of viral hepatitis, HIV and herpes simplex type 2 in the household population aged 21-64 years in Puerto Rico

**DOI:** 10.1186/1471-2334-10-76

**Published:** 2010-03-23

**Authors:** Cynthia M Pérez, Edmir Marrero, Marytere Meléndez, Sandra Adrovet, Héctor Colón, Ana P Ortiz, Marievelisse Soto-Salgado, Carmen Albizu, Esther A Torres, Erick Suárez

**Affiliations:** 1 Department of Biostatistics and Epidemiology, Graduate School of Public Health, Medical Sciences Campus, University of Puerto Rico, Puerto Rico; 2 Center for Sociomedical Research and Evaluation, Graduate School of Public Health, Medical Sciences Campus, University of Puerto Rico, Puerto Rico; 3 Cancer Control and Population Sciences Program, University of Puerto Rico Comprehensive Cancer Center, University of Puerto Rico, Puerto Rico; 4 Puerto Rico Cancer Center, School of Medicine, Medical Sciences Campus, University of Puerto Rico, Puerto Rico; 5 Department of Medicine, School of Medicine, Medical Sciences Campus, University of Puerto Rico, Puerto Rico

## Abstract

**Background:**

Viral hepatitis and sexually transmitted infections (STIs) are key public health problems that pose an enormous risk for disease transmission in the general population. This study estimated, for the first time, prevalence estimates of serologic markers of HCV, HBV, HAV, HIV and HSV-2 in the adult population of Puerto Rico and assessed variations across sociodemographic and behavioral characteristics.

**Methods:**

A seroepidemiologic survey was employed using a stratified cluster probability sample of households in Puerto Rico. Participants completed a face-to-face interview, a self-administered questionnaire using an ACASI system, and provided blood specimens for antibody testing. Prevalence estimates of viral hepatitis, HIV and HSV-2 were estimated using a logistic regression model weighting for the probability of participation in each household block and the inverse of the probability of selection according to geographic strata, households' blocks, and sex distribution.

**Results:**

A total of 1,654 adults participated in the study. Seroprevalence estimates for HCV (2.3%, 95% CI: 1.3%-4.2%), HBV (3.1%, 95% CI: 2.0%-4.7%), and HSV-2 (22.3%, 95% CI: 18.5%-26.7%) in Puerto Rico are roughly in agreement with estimates obtained in the US population; however, HAV (41.3%, 95% CI: 36.9%-45.8%) and HIV (1.1%, 95% CI: 0.5%-2.3%) seroprevalence estimates in Puerto Rico were higher. The proportion of individuals that were unaware of their serostatus was as follows: 80% for HCV, 98.3% for HBV, 96.4% for HAV, 36.4% for HIV, and 97.8% for HSV-2. Post-stratification estimates of seroprevalence varied significantly by demographic and risk related characteristics.

**Conclusion:**

This data underscore the disproportionate impact of some viral infections across selected population subgroups in Puerto Rico. A concerted island-wide effort is needed to strengthen viral hepatitis and STIs prevention and control strategies, support surveillance to monitor chronic infections, and ensure appropriate counseling, testing, and medical management of infected persons. Integration of HCV, HBV, and HSV-2 counseling into HIV existing prevention services and outreach through social networks might represent a valuable approach to reach high-risk individuals.

## Background

Viral hepatitis and sexually transmitted infections (STIs) are key public health threats that pose an enormous risk for disease transmission in the general population. Chronic infections with hepatitis B virus (HBV) and hepatitis C virus (HCV) are a leading cause of hepatocellular carcinoma, a condition whose incidence rate remains high in the developing world and is rising across most developed countries [1]. In 2007, the Centers for Disease Control and Prevention estimated that 85,000 new acute viral hepatitis cases and nearly 19 million new STIs occurred in the United States (US) [2,3]. In addition to the physical and psychological consequences of these diseases, the economic impact of STIs and of the viral hepatitis in the US are conservatively estimated at $15.3 billion annually, based on 2007 dollars, and $1.8 billion, based on 1999 dollars, respectively [4,5]. Taking into account that these statistics do not reflect the true burden of viral hepatitis caused by chronic infection with HBV and HCV, that many cases of notifiable acute hepatitis and STIs are undiagnosed, and some common viral infections, such as genital herpes and human papillomavirus infection, are not notifiable infectious diseases in the US, the true burden of viral hepatitis and STIs is underestimated [2,3,5,6].

Viral hepatitis and STIs share similar at-risk populations and risk factors. For example, those who are infected with certain STIs have a three to five-fold increased risk for HIV infection, 33% of all HIV-infected persons are HCV infected, and 5-15% of HIV-infected persons are co-infected with HBV [7,8]. These diseases also share similar social determinants, including poor access to health care, stigma, discrimination, homophobia, and poverty [5,6].

The National Health and Nutrition Examination Survey (NHANES) has provided reliable estimates of viral hepatitis (HCV, HBV, and hepatitis A virus (HAV)) and STIs including HIV and herpes simplex virus type 2 (HSV-2) across various racial and ethnic groups in the US [9-14]. However, Puerto Rico, as a jurisdiction, is excluded from NHANES. Accurate estimates for the impact of these viral infections in this Hispanic subgroup are important for predicting future trends and directing preventive efforts. Although the HIV/AIDS epidemic has disproportionately affected Puerto Ricans, with estimated rates per 100,000 population of 223.0 for HIV and 354.7 for AIDS during 2007, population-based surveys on viral hepatitis and STIs in Puerto Rico are scant [15-17]. Information on the burden of HIV and other STIs in Puerto Rico has relied upon surveillance systems or respondents' self-reports of symptoms and diagnoses provided in the Behavioral Risk Factor Surveillance Survey (BRFSS). Meanwhile, epidemiologic data regarding the burden of viral hepatitis and related complications are limited in scope and comprehensiveness [17], limiting the planning of prevention interventions and monitoring their effectiveness to control these infections. We determined for the first time prevalence estimates of serologic markers of HCV, HBV, HAV, HIV and HSV-2 and assessed variations across sociodemographic and behavioral characteristics in the adult population of Puerto Rico.

## Methods

### Study Design and Sampling Procedures

Detailed descriptions of the survey design and recruitment have been published elsewhere [18,19]. Briefly, a seroepidemiologic survey was designed using a stratified, multistage, probability cluster sample of all households in Puerto Rico. To define the sampling frame, Puerto Rico was stratified on the basis of AIDS incidence rates among injecting drug users (IDUs) and adult population density. The configuration of the sample strata assumed that HCV seroprevalence would vary systematically across the four strata and that prevalence estimates would gain precision. Four strata were derived corresponding to municipalities with above and at or below median AIDS incidence rates among IDUs (9.9/100,000 population reported in Puerto Rico in 2002) and above and at or below median population density (486 per square mile). Statistics provided by the Puerto Rico AIDS Surveillance System [20] were used to determine the incidence rate of AIDS among IDUs in each municipality in Puerto Rico during 2002. An estimated sample size of 2,000 adults randomly selected among strata of equal sizes was calculated on the basis of an expected prevalence of HCV infection of 6.3%, 95% confidence level and precision level of 3% [17,21]. Due to the complex sampling design, the prevalence estimation was weighted according to the inverse of probability of selection and participation rate in each selected block. The probability of selection was defined as a function of the geographic strata, households' blocks, and sex distribution according to post-censal estimates in Puerto Rico.

Household individuals within each stratum were selected in four stages. In the first stage, census blocks were sorted by median age and median housing value to achieve adequate representation of disease burden across all age groups and socioeconomic levels, and a systematic random selection was made with probability proportional to the number of households. The second selection stage consisted of a random selection of one block within each census block group. In a third stage, one segment of approximately 25 consecutive households was randomly selected from each block. A total of 108 segments were selected comprising 3,487 occupied households. Enumerators were able to contact residents in 3,386 households (97.1%), and 2,123 of these households had at least one adult aged 21 to 64 years old. In the final stage, one eligible adult was randomly selected from each household and invited to participate in the study.

Participants were given an appointment to visit a mobile examination unit located in the vicinity of their homes. At the mobile examination unit, a formal informed consent procedure was conducted which provided participants with information of all study procedures. Upon formally consenting, participants were offered pretest counseling. Participants completed a face-to-face interview and then were asked to complete a self-administered questionnaire using an ACASI system implemented using QDS (Nova Research Co., Washington, D.C.). After interviewing, a certified phlebotomist collected blood specimens of 15-ml for HAV, HBV, HCV, HIV, and HSV-2 testing. Participants received a $50.00 financial compensation upon completion of the study procedures for their time effort in addition to educational materials on prevention of blood-borne infections, STIs and enteric infections. Participants, whose test results were positive, received post-counseling and referrals for follow-up medical evaluation. Field work was conducted between June 2005 and February 2008. All human subjects provided informed consent for this study under a protocol (number #A0420105) approved by the University of Puerto Rico Medical Sciences Campus institutional review board.

### Measures

Face-to-face interviews covered standard sociodemographic characteristics plus extensive information on medical diagnoses including other STIs (self-reported syphilis, gonorrhea, genital warts, and infection with genital *Chlamydia trachomatis*), tattooing and body piercing practices, knowledge on viral infections under study, and self-reports of HAV and HBV vaccination coverage. The ACASI system ascertained cigarette and alcohol use, lifetime and recent drug use, sex-related risk behaviors, and history of incarceration. Trained interviewers conducted the personal interview and were available to answer questions during the self-administered interview.

### Laboratory Analyses

Testing for antibody to HCV was performed with a chemiluminiscent immunoassay (ADVIA Centaur^®^, Siemens Healthcare Diagnostics, Deerfield, IL) and confirmation by recombinant immunoblot assay (Laboratory Corporation of America^® ^Holdings, Tampa, FL). HBV antibodies were assayed with a chemiluminiscent immunoassay (IMMULITE^® ^2000, Siemens Healthcare Diagnostics, Los Angeles, CA) for total hepatitis B core antibody (anti-HBc), hepatitis B surface antigen (HBsAg) and hepatitis B surface antibody (anti-HBs). HBV serologic testing results were classified as follows: past or present infection (HbsAg negative, anti-HBc positive and anti-HBs positive; HBsAg positive, anti-HBc positive and anti-HBs negative) and serologic evidence of vaccination (anti-HBs positive, HBsAg negative and anti-HBc negative). Total HAV antibodies were measured by a chemiluminiscent immunoassay (ADVIA Centaur^®^, Siemens Healthcare Diagnostics, Deerfield, IL). HIV-1 antibodies were assayed with commercial enzyme immunoassay (Evolis™ Bio-Rad Laboratories, Inc., Hercules, CA) and confirmed by Western blot (Laboratory Corporation of America^® ^Holdings, Tampa, FL). HSV-2 type-specific IgG was tested via enzyme immunoassay (Laboratory Corporation of America^® ^Holdings, Tampa, FL).

### Statistical Analysis

Prevalence estimates of viral hepatitis (HAV, HBV, HCV), HIV and HSV-2 were estimated using logistic regression models weighting for the probability of participation in each household block and the inverse of the probability of selection according to the geographic strata, households' blocks, and sex distribution in Puerto Rico. To account for the correlation between subjects selected from the same block segment, the parameters of this model were estimated using a generalized estimating equations approach [22]. Population estimates were generated by multiplying population counts obtained from the postcensal estimates of the civilian, noninstitutionalized population aged 21-64 years as of July 1, 2008 by the weighted prevalence estimate and rounded to the nearest thousand [23]. Data management and statistical analyses were performed using the statistical package Stata (Version 10.0, College Station, TX, USA).

## Results

### Characteristics of Study Population

Of the selected residents, 1,654 (77.9%) consented to participate in the face-to-face interview and provided blood samples suitable for antibody testing for viral infections. The age distribution of study participants (21-29 years: 22.2%; 30-39 years: 24.2%; 40-49 years: 26.3%; 50-64 years: 27.3%) was similar to that of the adult population of Puerto Rico according to the Census 2000 [24] (Table 1). Females were slightly overrepresented in that they made up 56.4% of the study sample. Nearly 75% reported to have completed high school, 66.2% were living below the poverty level (annual family income below $20,000), and 10.5% did not have health care coverage at the time of the interview. More than half (51.3%) reported first sexual intercourse before age 18, 58% reported 2-9 lifetime sexual partners, and 6.1% of men had a history of sexual contact with same-sex partners. Over one-third (35.5%) of participants reported lifetime non-injection drug use, whereas 1.5% reported injection drug use. Marijuana (29.3%), cocaine (14.9%), and heroin (6.5%) were the most commonly used drugs. Nearly 15% informed tattooing practices, 30.1% had a history of ear or other body piercing, 4.8% reported receipt of blood transfusions prior to 1992, 5.2% had a history of other STIs, and 8.2% had a history of imprisonment. Self-reported vaccination coverage for HAV, defined as having received two doses of the vaccine, was indicated by only 1.6% of participants, whereas 13.2% had serologic evidence of hepatitis B vaccination.

**Table 1:** Demographic and risk-related characteristics of 1,654 adults aged 21-64 in Puerto Rico, 2005-2008

Characteristics	Number^a^	Percent (%)
Age in years		
21-29	367	22.2
30-39	400	24.2
40-49	435	26.3
50-64	452	27.3
Sex		
Female	933	56.4
Male	721	43.6
Years of education		
≥ 12	1237	74.8
< 12	417	25.2
Annual family income		
≥ 20,000	515	33.8
< $20,000	1011	66.2
Health care coverage		
Private	746	45.1
Public	735	44.4
None	173	10.5
Age in years at first sexual intercourse		
< 12	42	2.6
12-17	762	48.7
≥ 18	762	48.7
Number of lifetime sexual partners		
0-1	336	21.9
2-9	890	58.0
≥ 10	308	20.1
Men who have sex with men		
Never	633	93.9
Ever	41	6.1
Lifetime drug use		
None	1042	63.0
Non-IDU	587	35.5
IDU	25	1.5
Type of substance use^b^		
Marijuana	484	29.3
Cocaine	246	14.9
Heroin	108	6.5
Tattooing practice		
Never	1409	85.2
Ever	244	14.8
Body piercing		
Never	1154	69.9
Ever	498	30.1
Blood transfusions prior to 1992		
Never	1574	95.2
Ever	80	4.8
History of other STIs^c^		
No	1568	94.8
Yes	86	5.2
History of imprisonment		
Never	1518	91.8
Ever	136	8.2
Self-reported HAV vaccination coverage		
No	1628	98.4
Yes	26	1.6
Serologic evidence of HBV vaccination		
No	1435	86.8
Yes	219	13.2

^a^Total may not equal the overall sample size because of non-response.

^b^Categories of substance use are not mutually exclusive.

^c^Self-reported history of syphilis, gonorrhea, genital warts, and chlamydia infection.

### Prevalence estimation

#### HCV

Weighted prevalence was 2.3% (95% CI: 1.3% - 4.2%), corresponding to 53,000 (95% CI: 30,000 - 95,000) HCV antibody-positive persons in Puerto Rico (Table 2). This prevalence increased to 2.7% in strata 3 and 4, municipalities with a high incidence of AIDS among injecting drug users. Most of these individuals (80%) were unaware of their HCV serostatus (data not shown). Although the peak in age-specific prevalence was observed among people aged 40-49 years (2.7%) and was lowest among those aged 50-64 years (1.3%), variations across age groups were not statistically significant (Table 3). Anti-HCV prevalence was significantly higher in men (4.0%) and those with public (4.1%) or no health insurance (2.9%) (p < 0.01 and p < 0.001, respectively). Prevalence did not vary according to education and annual family income. When high risk behaviors were examined, anti-HCV prevalence was significantly (p < 0.001) higher for those who had first sex before the age of 12 years (10.2%) and among persons reporting at least 10 lifetime sexual partners (5.5%) but not among men who had sex with men. Anti-HCV prevalence was significantly (p < 0.001) higher among persons who had ever injected drugs (76.1%) compared to those who had ever used non-injection drugs (1.8%) or those who had never used drugs (0.6%). Anti-HCV was also significantly (p < 0.05) correlated with a history of tattooing practice (10.2%), body piercing (4.0%), receipt of blood transfusions before 1992 (6.3%), history of imprisonment (17.5%) but not with a history of other STIs. Positivity to HBV (17.4%) and HSV-2 (5.1%) antibody testing were significantly (p < 0.01) associated to HCV seropositivity (Table 4).

**Table 2:** Weighted seroprevalence (%) of selected viral infections among adults 21-64 years: Puerto Rico, 2005-2008

	Weighted prevalence of antibodies		Estimated number of adults infected in Puerto Rico^a^	
	Prevalence	95% CI	Number	95% CI
HCV (n = 1,650)	2.3	1.3 - 4.2	53000	30000 - 95000
Stratum 1^b^	2.1	1.1 - 4.1	9000	5000 - 17000
Stratum 2^c^	0.5	0.1 - 2.1	3000	1000 - 10000
Stratum 3^d^	2.7	1.6 - 4.8	8000	5000 - 13000
Stratum 4^e^	2.7	1.5 - 4.7	30000	17000 - 52000
HBV (n = 1,654)	3.1	2.0 - 4.7	71000	47000 - 108000
Stratum 1^b^	5.3	3.6 - 7.8	23000	15000 - 33000
Stratum 2^c^	2.6	1.4 - 4.6	12000	7000 - 22000
Stratum 3^d^	2.8	1.5 - 5.1	8000	5000 - 14000
Stratum 4^e^	2.9	1.7 - 5.0	33000	19000 - 56000
HAV (n = 1,650)	41.3	36.9 - 45.8	933000	834000 - 1035000
Stratum 1^b^	42.5	37.9 - 47.3	178000	159000 - 198000
Stratum 2^c^	38.5	33.9 - 43.3	180000	158000 - 202000
Stratum 3^d^	42.4	37.6 - 47.4	113000	100000 - 126000
Stratum 4^e^	42.0	37.2 - 46.8	465000	413000 - 520000
HIV (n = 1,650)	1.1	0.5 - 2.3	25000	12000 - 51000
Stratum 1^b^	0.2	0.03 - 1.4	1000	1000 - 6000
Stratum 2^c^	0.2	0.03 - 1.6	2000	1000 - 8000
Stratum 3^d^	0.7	0.2 - 2.1	2000	1000 - 6000
Stratum 4^e^	1.3	0.6 - 3.0	15000	7000 - 34000
HSV-2 (n = 1,446)	22.3	18.5 - 26.7	505000	418000 - 603000
Stratum 1^b^	19.7	16.0 - 24.1	83000	67000 - 101000
Stratum 2^c^	22.0	18.1 - 26.5	103000	85000 - 124000
Stratum 3^d^	24.9	20.5 - 29.8	66000	55000 - 80000
Stratum 4^e^	22.1	18.1 - 26.7	246000	201000 - 297000

^a^Estimated numbers and their associated 95% CI are rounded to the nearest thousand.

^b^Strata 1 include municipalities with low AIDS incidence rates among IDUs and low population density. ^c^Strata 2 include municipalities with low AIDS incidence rates among IDUs and high population density. ^d^Strata 3 include municipalities with high AIDS incidence rates among IDUs and low population density. ^e^Strata 4 include municipalities with high AIDS incidence rates among IDUs and high population density.

**Table 3:** Weighted seroprevalence of viral infections by selected sociodemographic characteristics and risk behaviors: Puerto Rico, 2005-2008.

Characteristics	Prevalence (%) (95% CI)				
	Anti-HCV	Anti-HBV	Anti-HAV	Anti-HIV	Anti-HSV-2
Age in years					
21-29	2.3 (0.8 - 6.6)	1.2 (0.4 - 3.3)^a^	8.2 (5.1 - 12.7)^c^	*^b, d^	14.8 (10.5 - 20.3)^c^
30-39	2.5 (1.2 - 4.9)	2.7 (1.3 - 5.4)	13.9 (9.9 - 19.2)	1.0 (0.3 - 3.5)	15.5 (10.7 - 21.8)
40-49	2.7 (1.3 - 5.4)	3.6 (2.1 - 6.2)	49.4 (41.7 - 57.2)	1.7 (0.6 - 4.7)	29.1 (24.2 - 34.5)
50-64	1.3 (0.4 - 4.4)	5.4 (3.3 - 8.6)	89.0 (84.6 - 92.2)	0.7 (0.2 - 2.0)	29.0 (23.3 - 35.4)
Sex					
Female	1.0 (0.4 - 2.1)^b^	2.5 (1.6 - 3.9)	41.3 (36.7 - 46.0)	0.8 (0.3 - 2.0)	25.6 (21.5 - 30.2)^b^
Male	4.0 (2.3 - 6.8)	4.3 (2.7 - 6.8)	41.5 (36.7 - 46.5)	1.0 (0.4 - 2.4)	17.8 (14.3 - 22.1)
Years of education					
≥ 12	2.2 (1.3 - 3.6)	2.5 (1.7 - 3.9)^a^	35.8 (32.3 - 39.4)^c^	0.6 (0.2 - 1.5)	20.2 (17.2 - 23.5)^b^
< 12	2.3 (1.0 - 5.3)	5.7 (3.7 - 8.6)	59.3 (54.5 - 63.9)	1.7 (0.7 - 4.3)	29.3 (22.8 - 36.8)
Annual family income					
≥ $20,000	1.4 (0.4 - 4.4)	1.1 (0.5 - 2.6)^b^	32.9 (28.6 - 37.5)^c^	1.1 (0.4 - 3.4)	16.9 (12.8 - 21.9)^b^
< $20,000	2.3 (1.3 - 4.0)	4.0 (2.9 - 5.6)	45.9 (41.8 - 50.1)	0.6 (0.2 - 1.6)	25.9 (21.8 - 30.5)
Health care coverage					
Private	0.4 (0.2 - 0.9)^c^	2.2 (1.3 - 3.7)	43.8 (38.9 - 48.9)	0.8 (0.2 - 2.3)	17.3 (14.0 - 21.3)^c^
Public	4.1 (2.4 - 6.9)	4.5 (3.0 - 6.7)	40.6 (36.0 - 45.3)	0.8 (0.3 - 1.9)	27.6 (23.0 - 32.6)
None	2.9 (0.8 - 9.6)	3.4 (1.4 - 8.2)	33.9 (24.2 - 45.1)	1.6 (0.3 - 7.8)	24.0 (16.5 - 33.6)
Age at first sexual intercourse					
< 12	10.2 (3.3 - 27.2)^c^	15.2 (5.7 - 34.5)^b^	57.2 (38.8 - 73.8)	2.0 (0.5 - 8.4)	33.1 (18.7 - 51.6)
12-17	3.6 (2.0 - 6.1)	3.7 (2.4 - 5.7)	38.3 (33.9 - 42.9)	0.9 (0.4 - 2.3)	23.9 (19.4 - 29.2)
≥ 18	0.5 (0.2 - 1.1)	1.9 (1.1 - 3.3)	43.2 (38.9 - 47.6)	0.3 (0.1 - 1.6)	20.1 (16.4 - 24.4)
Number of lifetime sexual partners					
0-1	0.7 (0.1 - 3.3)^c^	1.9 (0.9 - 4.0)	50.9 (44.3 - 57.6)^b^	*^d^	11.5 (7.9 - 16.5)^c^
2-9	1.2 (0.6 - 2.6)	2.7 (1.8 - 4.2)	37.4 (33.5 - 41.4)	0.9 (0.4 - 2.3)	24.1 (20.1 - 28.6)
≥ 10	5.5 (3.0 - 9.8)	4.6 (2.6 - 8.0)	41.5 (33.2 - 50.3)	0.7 (0.2 - 1.9)	25.1 (19.9 - 31.1)
Men who have sex with men					
Never	4.0 (2.3 - 6.9)	4.3 (2.6 - 7.1)	41.2 (36.3 - 46.2)	0.3 (0.1 - 0.9)^c^	16.8 (12.9 - 21.5)
Ever	2.6 (0.7 - 9.3)	3.1 (0.7 - 13.0)	49.9 (31.9 - 67.9)	7.3 (1.9 - 24.5)	22.0 (11.5 - 38.1)
Lifetime drug use					
None	0.6 (0.2 - 1.6)^c^	2.6 (1.7 - 3.9)^c^	47.7 (44.1 - 51.4)^c^	0.7 (0.3 - 1.9)	20.3 (16.6 - 24.5)
Non-IDU	1.8 (0.9 - 3.5)	3.5 (2.1 - 5.8)	29.3 (24.3 - 34.9)	1.0 (0.4 - 2.8)	25.4 (20.9 - 30.6)
IDU	76.1 (55.2 - 89.1)	23.5 (8.8 - 49.3)	66.9 (42.9 - 84.5)	2.8 (0.6 - 12.4)	31.9 (10.3 - 65.5)
Tattooing practice					
Never	0.9 (0.5 - 1.6)^c^	2.9 (2.0 - 4.0)	43.7 (40.4 - 47.0)^c^	0.6 (0.3 - 1.4)	21.8 (18.2 - 25.8)
Ever	10.2 (6.0 - 16.8)	5.6 (2.8 - 11.1)	27.5 (21.4 - 34.6)	2.2 (0.6 - 7.1)	26.2 (19.8 - 33.9)
Body piercing					
Never	1.4 (0.8 - 2.6)^a^	3.6 (2.5 - 5.1)	48.9 (45.2 - 52.7)^c^	1.0 (0.5 - 2.2)	23.0 (20.0 - 26.4)
Ever	4.0 (2.1 - 7.5)	2.7 (1.3 - 5.6)	24.4 (19.9 - 29.6)	0.5 (0.1 - 2.1)	21.0 (15.8 - 27.5)
Blood transfusions prior to 1992					
Never	2.0 (1.2 - 3.2)^b^	2.9 (2.1 - 4.0)^b^	40.1 (37.1 - 43.3)^c^	0.4 (0.2 - 1.0)^c^	21.9 (18.6 - 25.6)
Ever	6.3 (2.5 - 14.6)	11.6 (5.3 - 23.6)	65.2 (52.7 - 75.8)	9.2 (3.4 - 22.8)	31.2 (19.6 - 45.7)
History of other STIs^e^					
Yes	2.7 (0.7 - 9.1)	4.9 (2.2 - 10.7)	47.3 (35.0 - 59.8)	5.9 (1.9 - 16.5)^b^	33.9 (22.7 - 47.3)^a^
No	2.2 (1.3 - 3.6)	3.2 (2.3 - 4.5)	41.1 (37.9 - 44.3)	0.6 (0.3 - 1.4)	21.9 (18.6 - 25.6)
History of imprisonment					
Never	0.8 (0.4 - 1.5)^c^	2.6 (1.8 - 3.7)^b^	41.2 (37.8 - 44.7)	0.6 (0.2 - 1.3)^b^	21.1 (18.1 - 24.5)^b^
Ever	17.5 (11.4 - 26.1)	11.2 (5.4 - 21.7)	43.0 (33.5 - 53.0)	4.1 (1.4 - 10.9)	36.0 (25.4 - 48.0)

^a^P < 0.05; ^b^P < 0.01; ^c^P < 0.001.

^d^Indicates a relative standard error of 30% or more, thus this reflects a potentially unreliable estimate and is not shown.

^e^Self-reported history of syphilis, gonorrhea, genital warts, and chlamydia infection.

**Table 4:** Prevalence of concomitant antibodies to viral hepatitis, HIV and HSV-2, Puerto Rico, 2005-2008.

	Prevalence (%) (95% CI)				
Characteristic	Anti-HCV	Anti-HBV	Anti-HAV	Anti-HIV	Anti-HSV-2
Antibody to HCV					
Positive	--	27.1 (13.9 - 46.0)^a^	61.2 (34.5 - 82.5)	2.2 (0.5 - 8.6)	55.4 (30.9 - 77.6)^b^
Negative	--	2.7 (2.0 - 3.8)	40.9 (37.8 - 44.1)	0.8 (0.4 - 1.6)	21.7 (18.5 - 25.3)
Antibody to HBV					
Positive	17.4 (8.5 - 32.3)^a^	--	71.5 (56.7 - 82.7)^a^	--	39.9 (27.5 - 53.7)^b^
Negative	1.7 (1.0 - 2.8)	--	40.3 (37.1 - 43.6)	--	21.7 (18.6 - 25.3)
Antibody to HAV					
Positive	3.3 (1.8 - 6.1)	5.7 (3.9 - 8.3)^a^	--	1.4 (0.6 - 3.0)	29.4 (24.8 - 34.5)^a^
Negative	1.4 (0.6 - 3.2)	1.6 (1.0 - 2.7)	--	0.5 (0.1 - 1.7)	17.6 (14.2 - 21.5)
Antibody to HIV					
Positive	5.2 (1.0 - 23.0)	--	66.2 (29.5 - 90.1)	--	89.3 (57.0 - 98.1)^a^
Negative	2.2 (1.3 - 3.5)	--	41.1 (37.9 - 44.4)	--	21.9 (18.7 - 25.4)
Antibody to HSV-2					
Positive	5.1 (2.5 - 10.1)^b^	6.2 (3.9 - 9.8)^b^	53.7 (46.5 - 60.8)^a^	3.0 (1.2 - 6.9)^a^	--
Negative	1.2 (0.6 - 2.3)	2.7 (1.8 - 3.9)	36.9 (33.9 - 40.0)	0.1 (0.02 - 0.4)	--

--, Not applicable.

^a^P < 0.001; ^b^P < 0.01.

#### HBV

Weighted prevalence of past or present infection with HBV was 3.1% (95% CI: 2.0% - 4.7%), equating to 71,000 (95% CI: 47,000 - 108,000) adults with past or present HBV infection in Puerto Rico (Table 2). Prevalence of past or present infection with HBV was similar across strata. The majority of HBV seropositive individuals (98.3%) were unaware of their serostatus (data not shown). Prevalence of past or present HBV infection increased significantly (p < 0.05) with age, reaching 5.4% among individuals aged 50-64 years (Table 3). Although prevalence was higher in males (4.3%) and among those with public (4.5%) or no health insurance (3.4%), the differences were marginally significant (p = 0.07 and p = 0.097, respectively). Prevalence was significantly (p < 0.05) higher in those with less than 12 years of education (5.7%), those living below the poverty level (4.0%), those who had first sex before the age of 12 years (15.2%), persons who had ever injected drugs (23.5%), and those with a history of receiving blood transfusions before 1992 (11.6%) and imprisonment (11.2%). People with positive antibody testing results for HCV (27.1%), HAV (5.7%) and HSV-2 (6.2%) had significantly (p < 0.01) higher HBV seroprevalence than those with negative results (Table 4).

#### HAV

Weighted prevalence of anti-HAV was 41.3% (95% CI: 36.9% - 45.8%), and the estimated number of adults ever infected with HAV in Puerto Rico was 933,000 (95% CI: 834,000 - 1,035,000) (Table 2). No marked variations in HAV seroprevalence were observed across strata. Most of HAV seropositive individuals (96.4%) were unaware of their serostatus (data not shown). Prevalence of anti-HAV increased steeply (p < 0.001) with age, from 8.2% in persons aged 21-29 years to 89.0% in those aged 50-64 years (Table 3). Similarly, prevalence was significantly (p < 0.001) higher in people who had less than 12 years of education (59.3%) and lower annual family income (45.9%). Moreover, anti-HAV prevalence was higher (p < 0.01) among those who reported either one or no sexual partner (50.9%), injection drug use (66.9%), no history of tattooing or body piercing practices (43.7% and 48.9%, respectively), and receipt of blood transfusions prior to 1992 (65.2%). Individuals with positive results on HBV (71.5%) and HSV-2 (53.7%) antibody testing had significantly (p < 0.001) higher seroprevalence of HAV than seronegative individuals (Table 4).

#### HIV

Weighted prevalence was 1.1% (95% CI: 0.5% - 2.3%), corresponding to 25,000 (95% CI: 12,000 - 51,000) persons ever infected with HIV in the adult population of Puerto Rico (Table 2). This prevalence reached 1.3% in stratum 4 (municipalities with a high population density and high AIDS incidence rate among IDUs). The percentage of HIV seropositive individuals who were unaware of their status was 36.4% (data not shown). Prevalence of HIV was significantly (p < 0.05) higher in adults aged 40-49 years (1.7%), men who had sex with men (7.3%), those who reported receipt of blood transfusions prior to 1992 (9.2%), and individuals with a history of other STIs (5.9%) and imprisonment (4.1%) (Table 3). Adults who were seropositive to HSV-2 had a significantly higher prevalence of HIV antibodies than those who were seronegative (3.0% vs. 0.1%, p < 0.001) (Table 4).

#### HSV-2

Weighted prevalence was 22.3% (95% CI: 18.5% - 26.7%), equating to 505,000 (95% CI: 418,000 - 603,000) seropositive individuals to HSV-2 in the adult population of Puerto Rico (Table 2). Most of these individuals (97.8%) were unaware of their HSV-2 serostatus (data not shown). Seroprevalence was significantly (p < 0.01) higher among adults aged 40-49 and 50-64 years (29.1% and 29.0%, respectively) and females (25.6%) (Table 3). Prevalence was also significantly (p < 0.01) higher among those who had less than 12 years of education (29.3%), lived below the poverty level (25.9%), and were covered by public health insurance or were uninsured (27.6% and 24.0%, respectively). HSV-2 seropositivity significantly (p < 0.001) increased with the lifetime number of sexual partners, from 11.5% for one partner or less to 25.1% for those with at least 10 sexual partners. Individuals who reported a history of other STIs and those with a history of imprisonment were also significantly more likely to be HSV-2 seropositive than those without such histories (33.9% vs. 21.9%, p < 0.05; 36.0% vs. 21.1%, p < 0.01; respectively). People with positive results for anti-HCV (55.4%), anti-HBV (39.9%), anti-HAV (29.4%), and anti-HIV (89.3%) had significantly (p < 0.01) higher seroprevalence of HSV-2 compared to those with negative results (Table 4).

## Discussion

To our knowledge, this is the first population-based survey that provides nationally representative estimates of the seroprevalence of viral hepatitis, HIV and HSV-2 in the non-institutionalized adult population of Puerto Rico, vital information that provides insight into disease burden and opportunities for prevention. Seroprevalence estimates for HCV (2.3%), HBV (3.1%), and HSV-2 (22.3%) in Puerto Rico are roughly in agreement with estimates obtained in the US population aged 20-59 years, where age-adjusted prevalence was 2.4% for HCV (1.6% in the most recent NHANES), 5.6% for HBV, and 25.1% for HSV-2 [9-12]. However, HIV and HAV seroprevalence estimates in Puerto Rico (1.1% and 41.3%, respectively) were higher than the corresponding figures for the US adult population (0.5% and 30.7%, respectively) [13,14]. The higher HIV seroprevalence found in this study is consistent with national surveillance data showing that Puerto Rico has one of the highest overall rates of HIV/AIDS among states and territories of the US, mainly attributed to sharing of contaminated injecting equipment [15,16]. The higher HAV seroprevalence might be attributed to poorer economic development, sanitation, and environmental conditions in Puerto Rico [13,25]. However, prevalence estimates for viral hepatitis, HIV and HSV-2 in these studies are probably underestimated because the sampling frame excluded subpopulations at high risk of these infections including the homeless and incarcerated individuals [26,27].

The vast majority of individuals first learned of their viral hepatitis and HSV-2 serostatus from this survey. These findings parallel results from other studies that have consistently found that a large percentage of the population is not aware of their serostatus due to the asymptomatic nature of these infections [11,28]. The percentage of people unaware of their HIV seropositivity (36%), although substantially lower than for the other viral infections under study, is higher than the estimate in the US, where approximately 21% of HIV-positive persons remain undiagnosed [29]. This finding calls for a multisectoral approach to the HIV/AIDS epidemic in Puerto Rico to improve counseling, testing, and linkage to appropriate care and treatment services among high-risk individuals. In addition, widespread implementation of prevention interventions with proven efficacy could further prevent transmission of HIV [30]. Taking into consideration that the prevalence of antibodies to HCV, HBV and HSV-2 were considerably higher than the prevalence of HIV, the need for effective approaches for the prevention and control of viral hepatitis and other STIs is underscored. To increase knowledge and awareness of these infections among health care providers, at-risk populations and the public, integration of HCV, HBV and HSV-2 counseling and screening into HIV existing prevention services and outreach through social, sexual and drug-using networks might represent a valuable approach to reach high-risk individuals [31].

Dramatic variations in the prevalence of antibodies to viral hepatitis, HIV and HSV-2 were observed according to demographic and behavioral factors. As expected, anti-HAV increased markedly with age, primarily reflecting infection in the older cohorts that were acquired during childhood [13,25]. Antibodies to both HBV and HSV-2 also increased with age, findings consistent with reports from different geographic areas [10,12,32]. HIV seroprevalence increased to a peak of 1.7% in those aged 40-49 years and decreased to 0.7% in those aged 50-64 years. These observations are also consistent with HIV surveillance data in Puerto Rico where the majority of new infections in 2006 occurred among persons aged 30-49 years [16].

Significant sex differences were found for seroprevalence of HCV and HSV-2. Men were four times more likely to be anti-HCV positive, a finding that is in agreement with several population-based surveys, possibly reflecting riskier drug behaviors in males [11,33]. On the other hand, prevalence of HSV-2 antibodies was significantly higher among women. An extensive review of the prevalence of HSV-2 serum antibodies for different populations worldwide documents a higher prevalence among women [31]. Potential explanations for this finding include the higher efficiency of HSV-2 transmission from men to women as compared with that from women to men and differences between women and men in sexual behaviors [10,31].

Prevalence of antibodies to viral hepatitis and HSV-2 was significantly higher in those with lower socioeconomic status in Puerto Rico, defined as having less than a high school education, living below the poverty level or having a public or no health insurance. Epidemiologic studies worldwide have shown an inverse association between HAV seropositivity and socioeconomic indicators, particularly in developing countries [13,25]. Similarly, studies show socioeconomic status, poverty and geography to be major determinants of STIs disparities [5,6].

In general, prevalence of viral infections was higher among higher risk sexual behavior groups. Although efficiency of sexual transmission of HCV appears to be relatively low, the dose-response relationships observed between prevalence of anti-HCV and age at first sexual relation and number of lifetime sexual partners is in agreement with other studies [2,11,17]. Recent surveillance data for acute viral hepatitis in the US during 2007 found that 42% of cases reported having multiple sex partners, 10% reported having sexual contact with another known HCV infected person, and 10% were men who had sex with men [2]. Prevalence of past or present HBV infection was higher for individuals who had their first sexual intercourse before the age of 12 years than among those whose first sex was later in life. HSV-2 seroprevalence for adults who reported having had at least two sexual partners was more than twice the seroprevalence for those who reported only one or no sexual partner in their lifetime. HIV seropositivity was significantly higher in men who ever reported sex with other men than in men who did not report same-sex contact. These observations are consistent with surveillance findings and previous seroepidemiologic surveys and support the notion that adoption of HIV and other STIs' risk reduction behaviors should be encouraged for the general population, especially those at high risk, including barrier precautions to reduce the risk for exposure to these viral agents [10,12,14,28,32].

There is ample evidence showing that injection drug use is the single most important risk factor for HCV infection. In this study, prevalence of anti-HCV was 76% among those who reported lifetime injection drug use. In addition, prevalence of HCV antibodies among those who reported lifetime non-injection drug use was three-fold higher than those who reported no drug use. Prevalence of anti-HCV among IDU was higher than the reported figure in the NHANES 1999-2002 (57.5%) and in the US surveillance system for acute viral hepatitis during 1998-2007 (average: 44%; range: 38%-54%) [2,11,17]. Injection drug use was also common among HBV and HAV seropositive individuals, findings consistent with surveillance data for acute viral hepatitis in the US during 2007 [2].

Several infectious diseases have been found to be associated with a history of cosmetic procedures, such as tattooing and body piercing [34,35]. In our study, individuals with a history of tattooing and body piercing practices had a higher HCV seroprevalence than those without such practices. Similarly, adults who reported a history of imprisonment were also more likely to be seropositive to HCV, HBV, HIV and HSV-2. These findings support the implementation of a comprehensive approach to prevent these infections in the correctional setting [36]. Further investigation is also required to determine if these types of exposures contribute to the transmission of blood borne pathogens in the general population.

The Centers for Disease Control and Prevention have estimated that blood transfusions might have been the source of HCV infection for nearly 7% of cases [2]. In our study, 6.3% of adults who had a history of receiving blood transfusions prior to 1992 were three-fold more likely to be HCV seropositive than individuals without such a history. Similarly, individuals who reported receipt of blood transfusions also had a higher prevalence of antibodies for HBV and HIV, possibly reflecting the high risk of acquiring these infections via transfused blood products prior to the introduction of more sensitive screening techniques to improve blood supply safety or from units donated during the antibody-negative window period before seroconversion [37].

A higher seroprevalence of HIV and HSV-2 was observed among adults who self-reported a history of other STIs including syphilis, gonorrhea, genital warts, and chlamydia infection. These findings are in agreement with seroprevalence surveys and surveillance data in the US, that show that the two most commonly reported STIs in the US, chlamydia and gonorrhea, along with syphilis and herpes, have been associated with an increased transmission of HIV [3,5-7]. Moreover, numerous observational studies suggest that genital herpes is associated with an increased risk of HIV acquisition and may account for 40% to 60% of new HIV infections in high HSV-2 prevalence populations [32,38]. When concomitant antibodies to viral infections under study were examined, individuals who were HSV-2 seropositive had a significantly higher prevalence of antibodies to HCV, HBV, HAV and HIV. Similarly, HBV seropositive individuals had a higher prevalence of antibodies to HCV and HAV. These findings support the view that viral hepatitis and STIs share similar at-risk populations and social determinants [5,7,8]. Continued efforts to prevent new infections in high risk groups are essential, along with expansion of target HAV and HBV vaccination programs, screening strategies for viral hepatitis and STIs, and preventive activities to reduce the progression in people with chronic hepatitis.

Strengths of our study are the inclusion of a probabilistic sample of adults which enhances the generalizability of the findings in Puerto Rico, an adequate response rate (78%), and extensive data regarding risk factors. This study also demonstrated the feasibility of conducting a household survey that combined face-to-face interviews, ACASI, and biochemical measurements in a mobile examination unit in Puerto Rico, a methodology similar to that used in the NHANES program. However, there are several limitations to be considered when interpreting the results of this study. Although the distribution of age, education level, and family income of our sample was comparable to that of the adult population of Puerto Rico, according to the US Census 2000 [24], females were overrepresented. However, prevalence of the viral infections under study was not affected when the corresponding proportions of males and females in Puerto Rico from the Census 2000 were considered. Second, the small number of HIV positive individuals limited our ability to make reliable prevalence estimates according to sociodemographic and behavioral characteristics. Third, prevalence of anti-HCV and anti-HBV does not distinguish between recent or remote infection, so these estimates do not shed light on the current burden of chronic viral hepatitis. Finally, other well-known risk factors for HCV infection, such as long-term hemodialysis, history of organ transplantation, and health care work involving frequent exposure to blood, were not evaluated because of their low frequency. However, CDC estimates that these risk factors generally account for fewer than 10% of HCV infections [2].

Notwithstanding these limitations, this study represents a further step toward a true understanding of the burden of these viral infections in Puerto Rico. Prevention and control of these infections is challenging given their asymptomatic nature. Immunization strategies have been developed to eliminate the spread of HBV and HAV infections. However, there are currently no licensed vaccines to prevent the acquisition of HCV, HIV and HSV-2, thus, prevention of these infections mostly depends on preventing high-risk injecting and sexual behaviors.

## Conclusions

This study sheds light on the epidemiology of viral hepatitis, HIV and HSV-2 infections and underscores their disproportionate impact among specific population subgroups in Puerto Rico. Further research and policy development are urgently needed to strengthen viral hepatitis and STIs prevention and control strategies, improve surveillance of these infections, and provide integrated health services to ensure appropriate counseling, testing, and medical management of infected persons.

## Competing interests

The authors declare that they have no competing interests.

## Authors' contributions

CMP and ES conceived and supervised the implementation of the study. HC, CA, APO and EAT contributed to study concept and design and provided technical assistance and problem solving in the execution of the study. EM, MM, SA and MMS were responsible for study recruitment, data acquisition and overseeing daily operations of the study. CMP drafted the manuscript, and ES was responsible for all statistical analyses. All authors revised the manuscript critically for important intellectual content and approved the final manuscript.

## Pre-publication history

The pre-publication history for this paper can be accessed here:

http://www.biomedcentral.com/1471-2334/10/76/prepub
